# 1,3,3,5-Tetra­methyl-1*H*-1,5-benzodiazepine-2,4(3*H*,5*H*)-dione

**DOI:** 10.1107/S1600536811007501

**Published:** 2011-03-05

**Authors:** Rachida Dardouri, Youssef Kandri Rodi, Natalie Saffon, El Mokhtar Essassi, Seik Weng Ng

**Affiliations:** a Laboratoire de Chimie Organique Appliquée, Faculté des Sciences et Techniques Université Sidi Mohamed Ben Abdallah, Fés, Morocco; b Service Commun Rayons-X FR2599, Université Paul Sabatier, Bâtiment 2R1, 118 route de Narbonne, Toulouse, France; c Laboratoire de Chimie Organique Hétérocyclique, Pôle de Compétences Pharmacochimie, Université Mohammed V-Agdal, BP 1014 Avenue Ibn Batout, Rabat, Morocco; d Department of Chemistry, University of Malaya, 50603 Kuala Lumpur, Malaysia

## Abstract

The seven-membered ring in the title compound, C_13_H_16_N_2_O_2_, adopts a boat-shaped conformation (with the C atoms of the fused-ring as the stern and the C atom bearing two methyl groups) as the prow.

## Related literature

For the crystal structure of 1,5-dimethyl-1,5-benzodiazepin-2,4-dione, see: Mondieig *et al.* (2005 ▶).
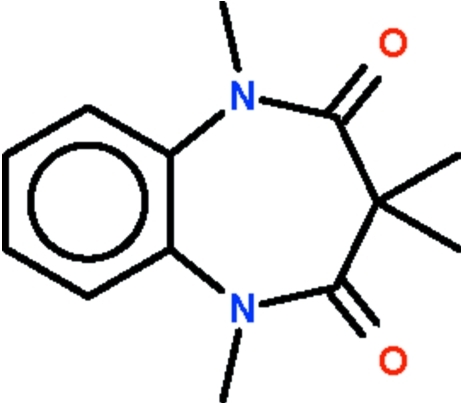


## Experimental

### 

#### Crystal data

C_13_H_16_N_2_O_2_
*M* *_r_* = 232.28
Monoclinic, 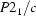

*a* = 7.5112 (1) Å
*b* = 10.1731 (2) Å
*c* = 15.8697 (3) Å
β = 103.675 (1)°
*V* = 1178.26 (4) Å^3^
*Z* = 4
Mo *K*α radiation
μ = 0.09 mm^−1^
*T* = 295 K
0.32 × 0.20 × 0.18 mm

#### Data collection

Bruker X8 APEXII diffractometer
20993 measured reflections
2719 independent reflections
2130 reflections with *I* > 2σ(*I*)
*R*_int_ = 0.042

#### Refinement

*R*[*F* ^2^ > 2σ(*F* ^2^)] = 0.039
*wR*(*F* ^2^) = 0.108
*S* = 1.04
2719 reflections
158 parameters
H-atom parameters constrained
Δρ_max_ = 0.26 e Å^−3^
Δρ_min_ = −0.20 e Å^−3^

### 

Data collection: *APEX2* (Bruker, 2008 ▶); cell refinement: *SAINT* (Bruker, 2008 ▶); data reduction: *SAINT*; program(s) used to solve structure: *SHELXS97* (Sheldrick, 2008 ▶); program(s) used to refine structure: *SHELXL97* (Sheldrick, 2008 ▶); molecular graphics: *X-SEED* (Barbour, 2001 ▶); software used to prepare material for publication: *publCIF* (Westrip, 2010 ▶).

## Supplementary Material

Crystal structure: contains datablocks global, I. DOI: 10.1107/S1600536811007501/bt5483sup1.cif

Structure factors: contains datablocks I. DOI: 10.1107/S1600536811007501/bt5483Isup2.hkl

Additional supplementary materials:  crystallographic information; 3D view; checkCIF report

## Figures and Tables

**Fig. 1.**
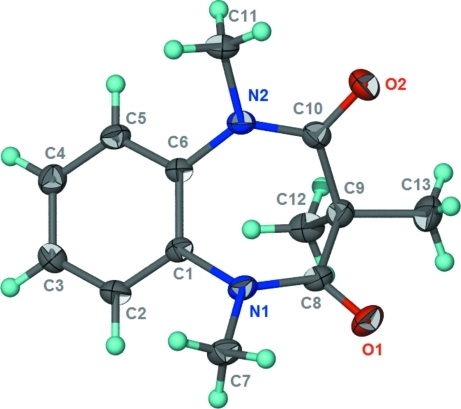
Thermal ellipsoid plot (Barbour, 2001) of C13H16N2O2 at the 50% probability level; hydrogen atoms are drawn as arbitrary radius.

**Fig. 2.**
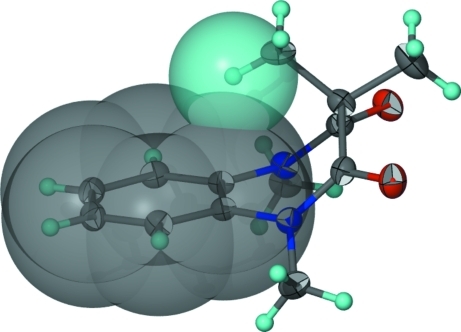
Thermal ellipsoid plot (Barbour, 2001) showing van der Waals surfaces for the carbon atoms of the phenylene ring as well as the van der Waals surface for one of the methyl hydrogen atoms.

## supplementary crystallographic information

### Comment

The methylene part of 1,5-dimethyl-1,5-benzodiazepine-2,4-dione is relatively acidic, and one proton can be abstracted by using potassium *t*-butoxide; the resulting carbanion can undergo a nucleophlilic subsitution with haloalkane to form 3-substituted derivatives. Previous studies have largely described the mono-substituted derivatives only. 1,12-Dibromodocane yielded the mono-substituted 12-bromodeyl derivative. In this study, the compound is reacted with methyl iodide to yield the di-methylated compound (Scheme I). The seven-membered ring adopts a boat-shaped conformation (with the C atoms of the fused-ring as the stern and the C atom bearing two methyl groups) as the prow (Fig. 1). The methyl group occupying the axial position hovers over the seven-membered ring, and the methyl group appears to be stopped from tipping over because of the *π*-system of the phenylene ring (Fig. 2).

### Experimental

To a solution of the potassium *t*-butoxide (0.42 g, 3.6 mmol) in DMF (15 ml) was added 1,5-dimethyl-1,5-benzodiazepine-2,4-dione (0.50 g, 2.4 mmol) and methyl iodide (0.68 g, 4.80 mmol). Stirring was continued for 24 h. The reaction was monitored by thin layer ch romatography. The mixture was filtered and the solution evaporated to give colorless crystals.

### Refinement

Carbon-bound H-atoms were placed in calculated positions (C—H 0.93–0.96 Å) and were included in the refinement in the riding model approximation, with *U*(H) set to 1.2–1.5*U*_eq_(C).

### Crystal data

**Table:**

C_13_H_16_N_2_O_2_	*F*(000) = 496
*M**_r_* = 232.28	*D*_x_ = 1.309 Mg m^−^^3^
Monoclinic, *P*2_1_/*c*	Mo *K*α radiation, λ = 0.71073 Å
Hall symbol: -P 2ybc	Cell parameters from 4653 reflections
*a* = 7.5112 (1) Å	θ = 2.4–32.5°
*b* = 10.1731 (2) Å	µ = 0.09 mm^−^^1^
*c* = 15.8697 (3) Å	*T* = 295 K
β = 103.675 (1)°	Block, colorless
*V* = 1178.26 (4) Å^3^	0.32 × 0.20 × 0.18 mm
*Z* = 4	

### Data collection

**Table:**

Bruker X8 APEXII diffractometer	2130 reflections with *I* > 2σ(*I*)
Radiation source: fine-focus sealed tube	*R*_int_ = 0.042
graphite	θ_max_ = 27.5°, θ_min_ = 2.4°
φ and ω scans	*h* = −9→9
20993 measured reflections	*k* = −13→13
2719 independent reflections	*l* = −20→20

### Refinement

**Table:**

Refinement on *F*^2^	Primary atom site location: structure-invariant direct methods
Least-squares matrix: full	Secondary atom site location: difference Fourier map
*R*[*F*^2^ > 2σ(*F*^2^)] = 0.039	Hydrogen site location: inferred from neighbouring sites
*wR*(*F*^2^) = 0.108	H-atom parameters constrained
*S* = 1.04	*w* = 1/[σ^2^(*F*_o_^2^) + (0.0537*P*)^2^ + 0.280*P*] where *P* = (*F*_o_^2^ + 2*F*_c_^2^)/3
2719 reflections	(Δ/σ)_max_ = 0.001
158 parameters	Δρ_max_ = 0.26 e Å^−^^3^
0 restraints	Δρ_min_ = −0.20 e Å^−^^3^

### Fractional atomic coordinates and isotropic or equivalent isotropic displacement parameters (Å^2^)

**Table:**

	*x*	*y*	*z*	*U*_iso_*/*U*_eq_	
O1	0.16162 (14)	0.16773 (10)	0.73262 (7)	0.0395 (3)	
O2	0.56946 (14)	0.16400 (10)	0.59503 (7)	0.0400 (3)	
N1	0.25294 (15)	0.37740 (11)	0.72907 (7)	0.0288 (3)	
N2	0.54245 (14)	0.37284 (11)	0.63540 (7)	0.0275 (2)	
C1	0.29905 (16)	0.49020 (12)	0.68579 (8)	0.0253 (3)	
C2	0.20477 (18)	0.60726 (13)	0.68935 (9)	0.0307 (3)	
H2	0.1123	0.6096	0.7193	0.037*	
C3	0.24611 (19)	0.71969 (14)	0.64933 (9)	0.0327 (3)	
H3	0.1835	0.7974	0.6535	0.039*	
C4	0.38090 (19)	0.71718 (13)	0.60286 (9)	0.0328 (3)	
H4	0.4079	0.7924	0.5750	0.039*	
C5	0.47450 (18)	0.60146 (13)	0.59851 (9)	0.0303 (3)	
H5	0.5643	0.5994	0.5670	0.036*	
C6	0.43742 (16)	0.48743 (12)	0.64028 (8)	0.0250 (3)	
C7	0.2047 (2)	0.39958 (15)	0.81279 (9)	0.0351 (3)	
H7A	0.2283	0.3211	0.8472	0.053*	
H7B	0.0771	0.4217	0.8027	0.053*	
H7C	0.2771	0.4704	0.8430	0.053*	
C8	0.22299 (17)	0.25474 (13)	0.69423 (9)	0.0289 (3)	
C9	0.26599 (18)	0.22616 (13)	0.60544 (9)	0.0313 (3)	
C10	0.47116 (18)	0.25147 (13)	0.61116 (8)	0.0288 (3)	
C11	0.73932 (18)	0.39067 (15)	0.64072 (10)	0.0350 (3)	
H11A	0.8050	0.3139	0.6661	0.052*	
H11B	0.7832	0.4660	0.6760	0.052*	
H11C	0.7582	0.4038	0.5836	0.052*	
C12	0.13980 (19)	0.30489 (16)	0.53176 (9)	0.0371 (3)	
H12A	0.1699	0.2846	0.4777	0.056*	
H12B	0.1566	0.3973	0.5433	0.056*	
H12C	0.0144	0.2818	0.5284	0.056*	
C13	0.2302 (2)	0.07970 (15)	0.58570 (12)	0.0487 (4)	
H13A	0.3049	0.0282	0.6313	0.073*	
H13B	0.2599	0.0586	0.5317	0.073*	
H13C	0.1033	0.0606	0.5817	0.073*	

### Atomic displacement parameters (Å^2^)

**Table:**

	*U*^11^	*U*^22^	*U*^33^	*U*^12^	*U*^13^	*U*^23^
O1	0.0423 (6)	0.0378 (6)	0.0421 (6)	−0.0102 (4)	0.0170 (5)	0.0065 (4)
O2	0.0431 (6)	0.0331 (5)	0.0469 (6)	0.0082 (4)	0.0170 (5)	−0.0008 (4)
N1	0.0306 (6)	0.0327 (6)	0.0261 (5)	−0.0038 (4)	0.0128 (5)	0.0013 (4)
N2	0.0234 (5)	0.0299 (6)	0.0312 (6)	0.0015 (4)	0.0104 (4)	0.0019 (4)
C1	0.0250 (6)	0.0291 (6)	0.0226 (6)	−0.0028 (5)	0.0071 (5)	0.0006 (5)
C2	0.0272 (6)	0.0367 (7)	0.0305 (7)	0.0017 (5)	0.0111 (5)	−0.0016 (5)
C3	0.0329 (7)	0.0306 (7)	0.0347 (7)	0.0053 (5)	0.0079 (6)	−0.0004 (5)
C4	0.0375 (7)	0.0294 (7)	0.0323 (7)	−0.0014 (5)	0.0096 (6)	0.0042 (5)
C5	0.0298 (6)	0.0344 (7)	0.0299 (6)	−0.0017 (5)	0.0134 (5)	0.0027 (5)
C6	0.0232 (6)	0.0285 (6)	0.0237 (6)	−0.0003 (5)	0.0064 (5)	−0.0002 (5)
C7	0.0373 (7)	0.0449 (8)	0.0271 (7)	−0.0061 (6)	0.0156 (6)	−0.0004 (6)
C8	0.0243 (6)	0.0331 (7)	0.0300 (6)	−0.0028 (5)	0.0077 (5)	0.0030 (5)
C9	0.0333 (7)	0.0306 (7)	0.0313 (7)	−0.0053 (5)	0.0106 (5)	−0.0029 (5)
C10	0.0326 (7)	0.0302 (7)	0.0252 (6)	0.0036 (5)	0.0102 (5)	0.0039 (5)
C11	0.0241 (6)	0.0429 (8)	0.0401 (8)	0.0028 (5)	0.0119 (6)	0.0001 (6)
C12	0.0315 (7)	0.0512 (9)	0.0277 (7)	−0.0038 (6)	0.0053 (6)	−0.0045 (6)
C13	0.0576 (10)	0.0360 (8)	0.0571 (10)	−0.0134 (7)	0.0227 (8)	−0.0103 (7)

### Geometric parameters (Å, °)

**Table:**

O1—C8	1.2248 (15)	C5—H5	0.9300
O2—C10	1.2214 (15)	C7—H7A	0.9600
N1—C8	1.3619 (17)	C7—H7B	0.9600
N1—C1	1.4217 (15)	C7—H7C	0.9600
N1—C7	1.4749 (16)	C8—C9	1.5457 (18)
N2—C10	1.3645 (17)	C9—C13	1.533 (2)
N2—C6	1.4199 (16)	C9—C10	1.5438 (18)
N2—C11	1.4722 (16)	C9—C12	1.544 (2)
C1—C2	1.3934 (18)	C11—H11A	0.9600
C1—C6	1.3992 (16)	C11—H11B	0.9600
C2—C3	1.3788 (19)	C11—H11C	0.9600
C2—H2	0.9300	C12—H12A	0.9600
C3—C4	1.3866 (19)	C12—H12B	0.9600
C3—H3	0.9300	C12—H12C	0.9600
C4—C5	1.3813 (19)	C13—H13A	0.9600
C4—H4	0.9300	C13—H13B	0.9600
C5—C6	1.3964 (17)	C13—H13C	0.9600
			
C8—N1—C1	125.39 (10)	O1—C8—N1	120.38 (12)
C8—N1—C7	116.99 (11)	O1—C8—C9	120.12 (12)
C1—N1—C7	116.84 (11)	N1—C8—C9	119.50 (11)
C10—N2—C6	124.90 (11)	C13—C9—C10	107.37 (12)
C10—N2—C11	116.72 (11)	C13—C9—C12	107.62 (12)
C6—N2—C11	117.33 (11)	C10—C9—C12	112.55 (11)
C2—C1—C6	118.99 (11)	C13—C9—C8	107.74 (11)
C2—C1—N1	119.09 (10)	C10—C9—C8	109.63 (11)
C6—C1—N1	121.92 (11)	C12—C9—C8	111.70 (11)
C3—C2—C1	121.22 (12)	O2—C10—N2	120.12 (12)
C3—C2—H2	119.4	O2—C10—C9	120.71 (12)
C1—C2—H2	119.4	N2—C10—C9	119.16 (11)
C2—C3—C4	120.13 (13)	N2—C11—H11A	109.5
C2—C3—H3	119.9	N2—C11—H11B	109.5
C4—C3—H3	119.9	H11A—C11—H11B	109.5
C5—C4—C3	119.11 (12)	N2—C11—H11C	109.5
C5—C4—H4	120.4	H11A—C11—H11C	109.5
C3—C4—H4	120.4	H11B—C11—H11C	109.5
C4—C5—C6	121.56 (12)	C9—C12—H12A	109.5
C4—C5—H5	119.2	C9—C12—H12B	109.5
C6—C5—H5	119.2	H12A—C12—H12B	109.5
C5—C6—C1	118.96 (11)	C9—C12—H12C	109.5
C5—C6—N2	118.71 (11)	H12A—C12—H12C	109.5
C1—C6—N2	122.33 (11)	H12B—C12—H12C	109.5
N1—C7—H7A	109.5	C9—C13—H13A	109.5
N1—C7—H7B	109.5	C9—C13—H13B	109.5
H7A—C7—H7B	109.5	H13A—C13—H13B	109.5
N1—C7—H7C	109.5	C9—C13—H13C	109.5
H7A—C7—H7C	109.5	H13A—C13—H13C	109.5
H7B—C7—H7C	109.5	H13B—C13—H13C	109.5
			
C8—N1—C1—C2	132.49 (13)	C7—N1—C8—O1	−1.24 (19)
C7—N1—C1—C2	−37.06 (17)	C1—N1—C8—C9	8.88 (19)
C8—N1—C1—C6	−48.03 (18)	C7—N1—C8—C9	178.41 (12)
C7—N1—C1—C6	142.42 (12)	O1—C8—C9—C13	−3.72 (18)
C6—C1—C2—C3	−0.31 (19)	N1—C8—C9—C13	176.63 (13)
N1—C1—C2—C3	179.18 (12)	O1—C8—C9—C10	−120.26 (13)
C1—C2—C3—C4	1.4 (2)	N1—C8—C9—C10	60.09 (16)
C2—C3—C4—C5	−1.0 (2)	O1—C8—C9—C12	114.27 (14)
C3—C4—C5—C6	−0.5 (2)	N1—C8—C9—C12	−65.38 (16)
C4—C5—C6—C1	1.5 (2)	C6—N2—C10—O2	167.89 (12)
C4—C5—C6—N2	−178.11 (12)	C11—N2—C10—O2	−0.06 (18)
C2—C1—C6—C5	−1.14 (18)	C6—N2—C10—C9	−12.78 (18)
N1—C1—C6—C5	179.38 (12)	C11—N2—C10—C9	179.28 (11)
C2—C1—C6—N2	178.50 (12)	C13—C9—C10—O2	4.75 (18)
N1—C1—C6—N2	−0.98 (18)	C12—C9—C10—O2	−113.51 (14)
C10—N2—C6—C5	−128.94 (13)	C8—C9—C10—O2	121.52 (13)
C11—N2—C6—C5	38.94 (17)	C13—C9—C10—N2	−174.59 (12)
C10—N2—C6—C1	51.42 (18)	C12—C9—C10—N2	67.16 (16)
C11—N2—C6—C1	−140.70 (12)	C8—C9—C10—N2	−57.81 (15)
C1—N1—C8—O1	−170.77 (12)		
